# Ezetimibe + simvastatin versus doubling the dose of simvastatin in high cardiovascular risk diabetics: a multicenter, randomized trial (the LEAD study)

**DOI:** 10.1186/1475-2840-9-20

**Published:** 2010-05-21

**Authors:** Gianluca Bardini, Carlo B Giorda, Antonio E Pontiroli, Cristina Le Grazie, Carlo M Rotella

**Affiliations:** 1 Unit of Endocrinology, Department of Clinical Pathophysiology, University of Florence, Viale Pieraccini 6, 50139, Florence, Italy; 2 Diabetes Unit ASL Turin 5 Chieri, Italy; 3 Univeristy of Milan, Milan, Italy and San Paolo Hospital, via A di Rudinì 8, 20142 Milan, Italy; 4 Medical Director, MSD, Centro Direzionale Milano Due, Palazzo Borromini, 20090 Segrate Milano, Italy

## Abstract

**Background:**

The primary goal of therapy in patients with hypercholesterolemia and coronary heart disease (CHD) is reducing low-density lipoprotein cholesterol (LDL-C). This was a multicenter, randomized, double-blind, double-dummy study in patients with type 2 diabetes mellitus (T2DM).

**Methods:**

Adult patients with T2DM and CHD (N = 93) on a stable dose of simvastatin 20 mg with LDL-C ≥ 2.6 mmol/L (100 mg/dL) and ≤ 4.1 mmol/L (160 mg/dL) were randomized to ezetimibe 10 mg plus simvastatin 20 mg (EZ + simva 10/20 mg) or simvastatin 40 mg for 6 weeks. Percent change in LDL-C, high-density lipoprotein cholesterol, and triglycerides was assessed.

**Results:**

EZ + simva 10/20 mg produced a significantly greater change from treated baseline compared with simvastatin 40 mg in LDL-C (-32.2% vs -20.8%; p < 0.01) and total cholesterol (-20.6% vs -13.2%; p < 0.01). A greater proportion of patients achieved LDL-C < 2.6 mmol/L with EZ + simva 10/20 mg than with simvastatin 40 mg, but this was not statistically significant (78.4% vs 60%; odds ratio = 2.81; p = 0.052). Changes in high-density lipoprotein cholesterol and triglycerides were similar between treatments. Both treatments were generally well-tolerated.

**Conclusions:**

These results demonstrate that EZ + simva 10/20 mg may provide a superior alternative for LDL-C lowering vs doubling the dose of simvastatin to 40 mg in hyperlipidemic patients with T2DM and CHD. In addition, the combination therapy may provide an alternative treatment for patients who require further LDL-C reduction than they can achieve with simvastatin 20 mg alone.

## Background

Coronary heart disease (CHD) prevention through management of modifiable risk factors is of great importance [1-3]. The International Task Force, working in cooperation with the International Atherosclerosis Society [4], identified patients with diabetes mellitus as very high risk for CHD, and the National Cholesterol Education Program Adult Treatment Panel (NCEP ATP) III [1] classified diabetes mellitus as a CHD risk equivalent. Type 2 diabetes mellitus (T2DM) has been shown to be associated with increased risk for CHD, which is the major cause of mortality in patients with T2DM [5].

The consensus conference report from the American Diabetes Association and American College of Cardiology Foundation [6] suggests 3-hydroxy-3-methyl-glutaryl-CoA reductase inhibitors (statins) as initial therapy for management of lipoprotein abnormalities in patients with cardiometabolic risk, including T2DM. The recommended low-density lipoprotein cholesterol (LDL-C) treatment target for patients with T2DM is < 2.6 mmol/L (< 100 mg/dL), with < 1.8 mmol/L (< 70 mg/dL) as an optional therapeutic target in the US for patients with T2DM and CHD [1,4,6]; and in Europe, the targets are similar: < 2.5 mmol/L (< 97 mg/dL) with an optional goal of < 2.0 mmol/L (< 77 mg/dL) [2]. A recent meta-analysis of 14 randomized trials with statins in primary and secondary prevention in diabetes demonstrated the substantial benefit of statins in reducing major cardiovascular events independent of pre-treatment concentration of LDL-C and similar to that observed in non-diabetic subjects [7]. Despite evidence for the benefit of statin treatment, a considerable number of European patients are not treated with lipid-lowering drugs [8], and specifically, statins are appreciably underused in diabetic patients in Italy [9]. In patients receiving treatment, a moderate dose of statin may not be sufficient to reach recommended LDL-C targets [10]. Significant LDL-C reductions have been observed with more intensive therapy using higher doses of statins, but not all patients tolerate high-dose statins [11]. For some patients, the incidence of abnormalities in liver function or myopathy may increase in a dose-dependant manner with this class of drugs [12]; and even with a high dose, some patients still do not meet treatment goals. Irrespective of dose, another important aspect in determining the response to lipid-lowering drugs is the balance between cholesterol synthesis and absorption. Inter-individual variability of response has been demonstrated both with statins, which reduce cholesterol synthesis in the liver, and with ezetimibe, which selectively blocks intestinal absorption of cholesterol by binding the Niemann-Pick C1-Like 1 (NPC1L1) receptor [13,14]. By targeting both mechanisms a greater reduction in LDL-C may be achieved. Statin therapy combined with ezetimibe may provide effects on lipids that complement and surpass those of high-dose statins [15].

Ezetimibe monotherapy reduces LDL-C by ~18%, with beneficial effects on total cholesterol, apolipoprotein B, and triglycerides, and is generally well tolerated compared with placebo [16]. When ezetimibe is co-administered with a statin, complementary effects on the lipid profile have been demonstrated, without significant impact on the tolerability profile of either drug [16,17]. A number of randomized studies have been published that prospectively assessed the efficacy of ezetimibe plus statin specifically in diabetic patients with and without CHD [18-21]. Ezetimibe/simvastatin (EZ + simva) combination or ezetimibe added to statin in T2DM patients not at target with statin monotherapy consistently demonstrated greater improvements in the lipid profile and a higher proportion of patients achieving LDL-C targets compared with doubling the dose of either simvastatin or atorvastatin [18-21]. In order to extend and confirm these results in a homogeneous population of patients attending outpatient diabetes clinics in Italy, this study compared the LDL-C lowering efficacy and the safety and tolerability of ezetimibe co-administered with simvastatin 20 mg vs doubling the dose of simvastatin to 40 mg after 6 weeks of treatment in patients diagnosed with T2DM and CHD.

## Methods

### Study design

This was a multicenter, randomized, parallel-group, double-blind, double-dummy placebo-controlled study conducted at 22 sites in Italy from July 2005 to February 2007. The protocol (Protocol 04037) was reviewed and approved by an Independent Ethics Committee at each participating center, and patients provided written informed consent prior to any study-related procedure being started. The study was conducted under the provisions of the Declaration of Helsinki and in accordance with the International Conference on Harmonization Consolidated Guideline on Good Clinical Practice.

### Study population

Men and women aged 18-75 years with T2DM [with fasting plasma glucose ≥ 7.0 mmol/L (126 mg/dL) and hemoglobin (Hb) A1c ≤ 9.0%] of at least 12 months duration and documented CHD (including stable angina with evidence of ischemia on exercise testing, history of myocardial infarction, percutaneous transluminal coronary angioplasty, atherothrombotic cerebrovascular disease, unstable angina or non-Q wave myocardial infarction), or symptomatic peripheral vascular disease, who were taking a stable daily dose of simvastatin 20 mg for 6 weeks with good compliance (80% of daily doses for the 6 weeks prior to baseline visit) and had LDL-C concentration ≥ 2.6 mmol/L (100 mg/dL) to ≤ 4.1 mmol/L (160 mg/dL) were eligible for randomization. Patients were instructed to follow a healthy lifestyle (cholesterol-lowering diet and exercise) throughout the study. In addition, subjects were required to have triglyceride concentrations < 3.99 mmol/L (350 mg/dL), liver transaminases [alanine aminotransferase (ALT) or aspartate aminotransferase (AST)] and creatine phosphokinase (CK) < 50% above the upper limit of normal (ULN) with no active liver disease, and hematology, blood chemistry, and urinalysis within normal limits. Women of childbearing potential were required to use effective birth control.

Patients were excluded if they had Class III or IV congestive heart failure; uncontrolled cardiac arrhythmia; recent (within 3 months of randomization) myocardial infarction, acute coronary insufficiency, coronary artery bypass surgery, or angioplasty, unstable or severe peripheral artery disease; newly diagnosed or unstable angina pectoris, uncontrolled hypertension (treated or untreated); uncontrolled endocrine or metabolic disease known to influence serum lipids or lipoproteins; impaired renal function (creatinine > 2.0 mg/dL) or nephrotic syndrome; or were taking any lipid-lowering agents, fibrates, resins or niacins, or prescription and/or over-the-counter-drugs with the potential for significant lipid effects (other than study drug) or with potential drug interactions with the statins.

### Randomization and Blinding

Patients were randomized according to a computer-generated randomization schedule into two treatment sequences using a 1:1 ratio to receive either ezetimibe 10 mg + simvastatin 20 mg placebo or ezetimibe placebo + simvastatin 20 mg for 6 weeks. All patients continued the treatment with simvastatin 20 mg once daily using the open label simvastatin 20 mg supplied. Because the ezetimibe and simvastatin tablets were different in size, shape and color, this study used a double-blind, double-dummy design, i.e., ezetimibe and simvastatin tablets were administered together with the alternative placebo.

Blinding was maintained until after study completion and database closure. Patient compliance was assessed by tablet count returned at the end of study. Compliance < 70% was considered a major protocol violation.

### Efficacy measures

The primary efficacy measure was the percent change in LDL-C from baseline to endpoint after 6 weeks of treatment. Secondary efficacy measures were the percentage of patients who reached LDL-C ≤ 2.6 mmol/L (100 mg/dL) at endpoint and the percent change in total cholesterol, high-density lipoprotein cholesterol (HDL-C), and triglycerides from baseline to endpoint after 6 weeks of treatment. The basic lipid panel assessment was conducted at a central laboratory (Centro Diagnostico EXACTA, Verona, Italy). LDL-C measurements were calculated by the Friedewald equation [22].

### Safety and tolerability

Adverse events were monitored at each visit and summarized by system organ class and specific adverse experience term. Laboratory tests included complete blood count, total protein, albumin, calcium, inorganic phosphorus, fasting plasma glucose, blood urea nitrogen, uric acid, total bilirubin, alkaline phosphatase, ALT, AST, gamma glutamyl transpeptidase, serum creatinine, thyroid stimulating hormone (baseline only), HbA1c, sodium, potassium, chloride, CK; and urinalysis. The analysis of laboratory parameters was conducted at a cental laboratory (Centro Diagnostico EXACTA, Verona, Italy).

### Statistics

Primary and secondary endpoints were assessed in the intent-to-treat (ITT) population, which included all subjects who were randomized, had taken at least one dose of study drug, and had at least one measurement at baseline and after the start of treatment. The primary efficacy endpoint was assessed using the analysis of variance (ANOVA) model, which included terms for treatment effect. Estimates via least-squares means between mean differences and 95% confidence intervals were provided. Secondary efficacy parameters and laboratory variables were analyzed using ANOVA with treatment as a grouping factor. Multiple comparisons within and between treatments were performed. A stepwise logistic regression model with terms for treatment, baseline LDL-C, age, and HbA1c was used to predict the probability of achieving LDL-C < 2.6 mmol/L (100 mg/dL). The safety population included all randomized patients who took at least one dose of study drug. The incidence of adverse events was compared between treatments using the Fisher exact test with Yates correction if applicable.

## Results

**Figure 1:**
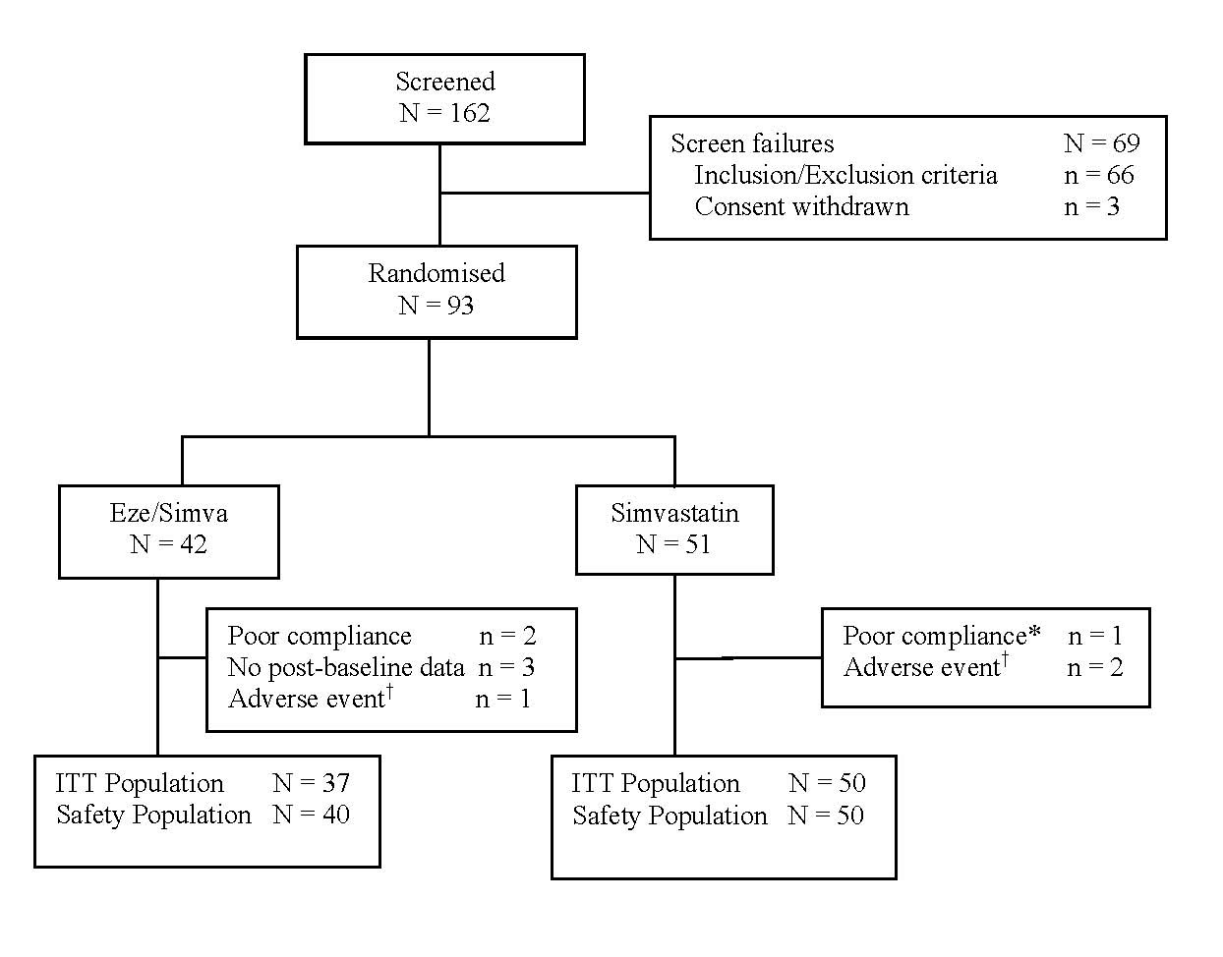
**Flow of patients through the study**. *No evidence of intake of study drug; ^†^Patients that discontinued the study due to adverse events underwent the final visit and were included in the ITT population.

The flow of patients through the study is shown in Figure 1. Of the 162 patients screened, 51 were randomized to the simvastatin group and 42 were randomized to the EZ + simva group. A total number of 6 patients withdrew from the study after randomization: 4 in the EZ + simva group and 2 in the simvastatin group. Reasons were adverse events in 1 patient and poor compliance in 3 in the EZ + simva group, and adverse events in the 2 patients in the simvastatin group. However, the 3 patients that discontinued the study due to adverse events underwent the final visit and were included in the ITT population. There were 3 patients with no evidence of intake of study drug, and these were excluded from the safety population.

Baseline characteristics are shown in Table 1. The mean age (± standard deviation) was 65 (± 6.5) years in the EZ + simva group and 64 (± 6.1) years in the simvastatin group. All patients were Caucasian and most patients were male (57% in the EZ + simva group and 76% in the simvastatin group; p = 0.078). The two treatment groups were similar with regard to demographic data, medical history, cardiovascular risk factors, and baseline values of all efficacy and safety parameters except AST (p = 0.001), ALT (p = 0.002), and CK (p = 0.002) mean values, which were significantly higher in the simvastatin group compared with the EZ + simva group. The mean LDL-C in the ITT population was 3.28 mmol/L (126.6 mg/dL) in the EZ + simva group and 3.24 mmol/L (125.2 mg/dL) in the simvastatin group. Table 1 shows the types of CHD reported at baseline. Ischemic heart disease was the most common form of CHD at baseline, with 22 (60%) patients in the EZ + simva group and 27 (54%) patients in the simvastatin group.

**Table 1:** Baseline patient characteristics

	EZ + Simva 10/20 mg (N = 37)	Simva 40 mg (N = 50)	Between treatment difference
**Demographics**			

Age, mean years (SD)	65 (6.5)	64 (6.1)	p = 0.204
Females, n (%)	16 (43.2)	12 (24.0)	p = 0.078
Body mass index, kg/m^2^, mean (SD)	28.9 (4.1)	28.4 (3.6)	p = 0.584
Hypertension, n (%)	30 (81.1)	32 (62.0)	p = 0.090

**Baseline lab values**, mean (SD)			

Low-density lipoprotein cholesterol, mmol/L	3.3 (0.5)	3.2 (0.5)	p = 0.413
Total cholesterol, mmol/L	5.2 (0.6)	5.1 (0.6)	p = 0.335
High-density lipoprotein cholesterol, mmol/L	1.2 (0.3)	1.1 (0.3)	p = 0.202
Triglycerides, mmol/L	1.6 (0.7)	1.6 (0.7)	p = 0.933
Fasting plasma glucose, mmol/L	10.2 (2.7)	10.0 (2.9)	p = 0.717
Hemoglobin A1c	7.5 (0.7)	7.4 (0.8)	p = 0.539
Aspartate aminotransferase, U/L	16.9 (3.4)	20.2 (5.1)	p = 0.001
Alanine aminotransferase, U/L	20.8 (7.1)	27.6 (11.4)	p = 0.002
Creatine kinase, U/L	80.4 (35.5)	107.5 (47.3)	p = 0.002

**Prevalence of cardiovascular diseases**, n (%)			

Cerebrovascular disease	3 (8.1)	5 (10.0)	p = 1.000
Peripheral vascular disease	7 (18.9)	11 (22.0)	p = 0.999
Ischemic heart disease	22 (59.5)	27 (54.0)	p = 0.666
Cerebrovascular disease + PAD	1 (2.7)	2 (4.0)	p = 1.000
Cerebrovascular disease + ischemic heart disease	0 (0.0)	0 (0.0)	--
PAD + ischemic heart disease	3 (8.1)	4 (8.0)	p = 1.000
Cerebrovascular disease + PAD + ischemic heart disease	1 (2.7)	1 (2.0)	p = 1.000

N, number in full analysis set population; SD, standard deviation; n, number; PAD, peripheral arterial disease

**Figure 2:**
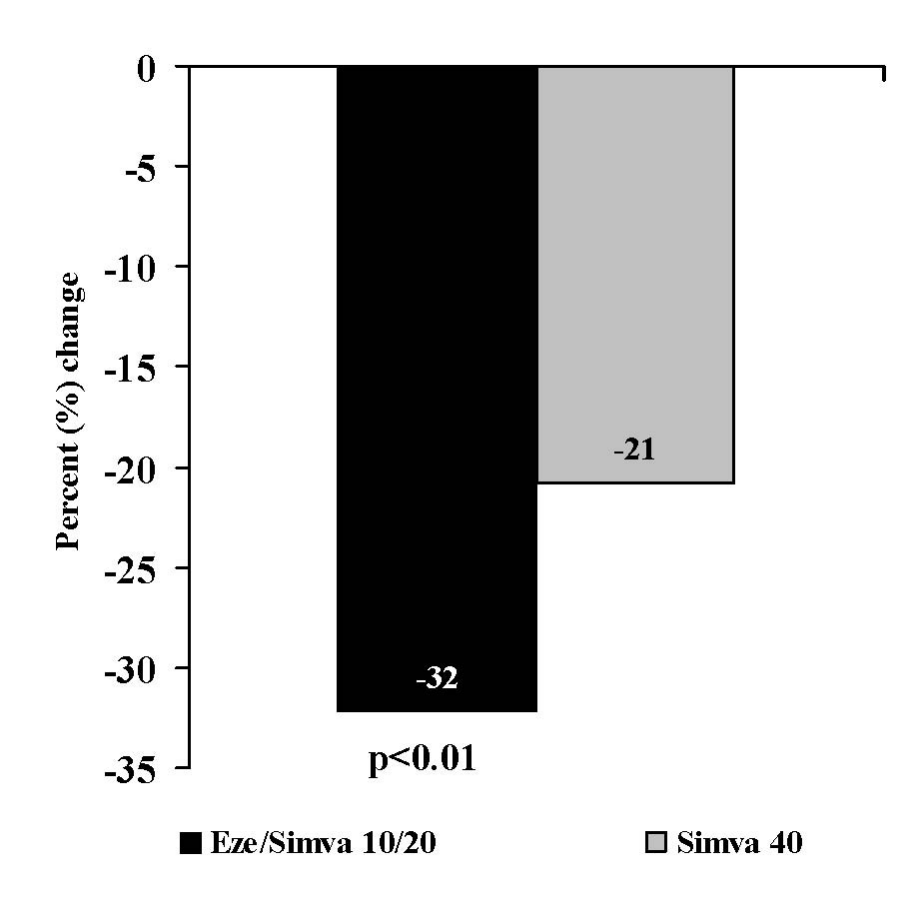
**Mean% change from baseline in LDL-C after 6 weeks of treatment**.

**Figure 3:**
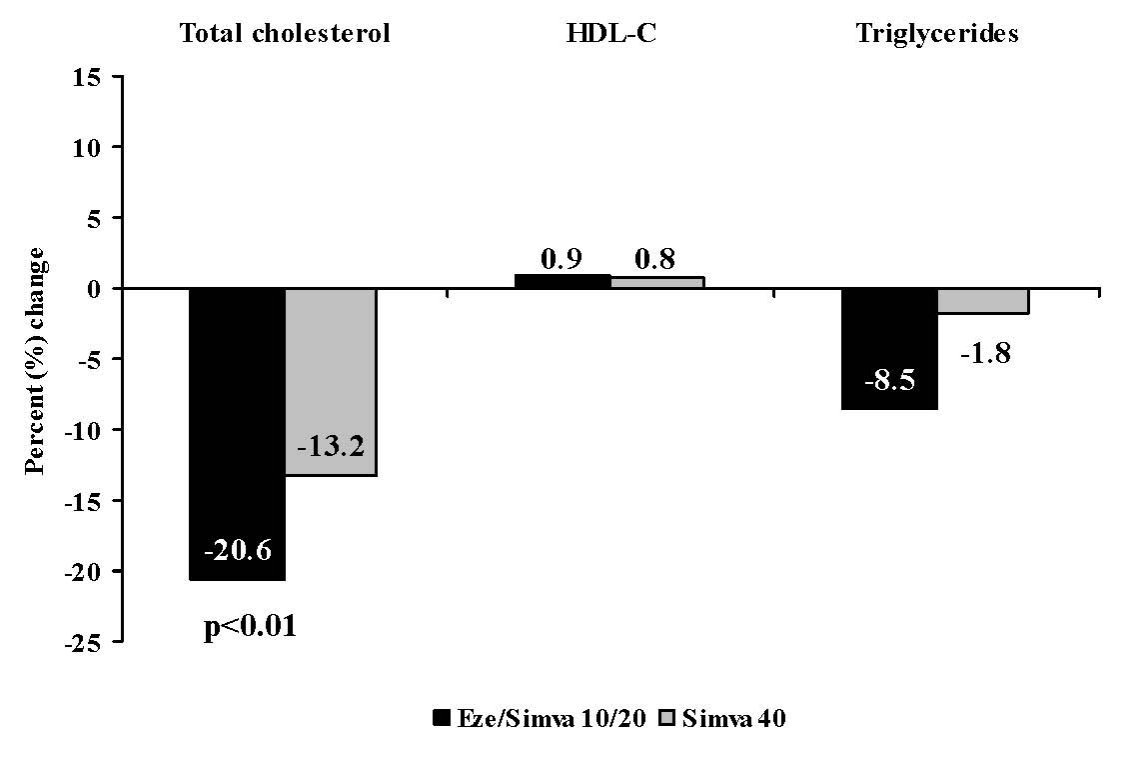
**Percent change in total cholesterol, HDL-C and triglycerides after 6 weeks of treatment**.

Compared with doubling the dose of simvastatin to 40 mg (Figure 2), treatment with EZ + simva 10/20 mg resulted in a significantly greater mean reduction in LDL-C after 6 weeks (-32.2% vs -20.8%; p < 0.01). In addition, the proportion of patients achieving the recommended LDL-C target < 2.6 mmol/L (< 100 mg/dL) at 6 weeks was numerically greater with EZ + simva 10/20 mg (78%) compared with simvastatin 40 mg treatment (60%), although this was not statistically significant (odds ratio = 2.81; 95% confidence interval: 0.99, 7.97, p = 0.052). The estimated treatment effect was dependent on baseline LDL-C value (p = 0.010), but not age or HbA1c level at baseline. The percent reduction in total cholesterol (Figure 3) was significantly greater after 6 weeks of EZ + simva 10/20 mg treatment compared with doubling the dose of simvastatin to 40 mg (-20.6% vs -13.2%; p < 0.01). Changes in HDL-C (0.85% EZ + simva vs 0.80% simvastatin) and triglycerides (-8.5% EZ + simva vs -1.8% simvastatin) were similar between treatment groups (Figure 3).

A summary of safety results is shown in Table 2. The proportion of patients who reported adverse events was similar between treatment groups (p = 0.507), with few discontinuations due to adverse events (only 1 patient in the EZ + simva group and 2 in the simvastatin group). No differences between groups were observed in the number and rate of drug-related events, which were reported in 7.5% of patients in the EZ + simva group and in 6.0% of patients in the simvastatin group (p = 1.000). One serious adverse event was reported (bone fracture) that was considered non-drug-related. There were no reports of increased ALT or AST ≥ 3 × ULN or CK ≥ 5 × ULN, and no deaths occurred at any time during the study in either treatment group. There were no clinically significant changes in plasma glucose or HbA1c in either group.

**Table 2:** Summary of safety data

	EZ + simva 10/20 mg	Simva 40 mg	p-value
**Number of patients (%)**	**N = 42**	**N = 51**	

With adverse events	5 (12.5)	10 (20.0)	0.40008
With treatment-related adverse events	3 (7.5)	3 (6.0)	0.9999
Discontinued due to adverse events	1 (2.5)	2 (4.0)	0.9999
Discontinued due to treatment-related adverse events	1 (2.5)	2 (4.0)	0.9999
Serious adverse events	1*	0	0.4518
ALT/AST ≥ 3 × upper limit of normal	0	0	--
CK ≥ 5-10 × upper limit of normal elevation	0	0	--

ALT, Alanine aminotransferase; AST, Aspartate aminotransferase; CK, creatine kinase.

*Bone fracture, non-drug-related.

## Discussion

These results demonstrated that ezetimibe 10 mg in combination with ongoing simvastatin 20 mg produced significantly greater LDL-C and total cholesterol reductions compared with doubling the dose of simvastatin to 40 mg in patients with primary hypercholesterolemia, T2DM, and CHD not at the LDL-C target < 2.6 mmol/L (< 100 mg/dL) with simvastatin 20 mg/day for at least 6 weeks. In addition, more patients in the combination EZ + simva 10/20 mg group achieved the target LDL-C goal as defined by the NCEP ATP III guidelines than in the simvastatin 40 mg group, although this was not statistically significant. The treatment effect was in favor of EZ + simva with nearly a threefold odds of reaching the LDL-C < 2.6 mmol/L (< 100 mg/dL) vs doubling the dose of simvastatin. The probability of reaching this target was independent of age and HbA1c level at baseline within the range of values of this population. Changes in HDL-C and triglycerides were similar between treatment groups, and these changes were consistent with results of previous trials comparing EZ + simva combination vs statin monotherapy in T2DM patients [19-21]. Treatment with EZ + simva 10/20 mg or simvastatin 40 mg was generally well-tolerated.

The results of this study were consistent with previous studies of similar design and duration conducted in the general population of patients with hypercholesterolemia and CHD [23,24]. They are also generally consistent in patients with T2DM [18-21,25]. In clinical studies of patients with T2DM not at the recommended LDL-C target < 2.6 mmol/L (< 100 mg/dL) with a previous statin treatment, EZ + simva was consistently superior to doubling the ongoing statin dose in reducing the LDL-C values and in getting patients to the LDL-C target [18,20,21]. Of note, in the present study the reduction in LDL-C that occurred with doubling the dose of simvastatin was higher than that observed in many previous studies of similar design, which showed reductions of < 10% [18,20,21]. These effects were also observed in a larger study with similar design to the present one comparing EZ + simva 10/20 mg with atorvastatin 20 mg in hypercholesterolemic CHD patients without diabetes [23]. The reasons for the inconsistency observed in the response to doubling statin dose are not fully understood. We speculate that this could be due to study effect or to good compliance. On the other hand, most studies and *post hoc *analyses did not include a comparison of goal attainment between patient groups, nor an analysis of factors that predict the odds of achieving goal. One report did show that more patients in the diabetes group achieved the recommended LDL-C goal compared with non-diabetics (83.6% versus 67.2%), although this result was not statistically significant after adjusting for differences in baseline LDL-C levels [26]. In addition, a significant interaction for LDL-C lowering was observed in a preliminary report of a *post hoc *analysis of patients in the IN-CROSS study, indicating larger between-group reductions in patients with T2DM versus those without T2DM [27]. Further study in larger populations that directly compare the efficacy of combination therapy with statin monotherapy in diabetics vs non-diabetics is warranted.

EZ + simva combination therapy at the usual recommended starting dose of 10/20 mg, and at the next higher dose of 10/40 mg vs the recommended usual starting dose and next highest dose of atorvastatin (10, 20, and 40 mg, respectively), was consistently superior to statin monotherapy in reducing LDL-C levels and attaining LDL-C levels < 1.8 mmol/L (< 70 mg/dL) in T2DM patients [19-21]. The results of the current trial confirm previous findings in patients with T2DM and extend them to include a population of patients treated in outpatient diabetes clinics.

Recent investigations on the changes in the cholesterol homeostasis in patients with CHD and/or diabetes seem to support the concept that a complementary approach targeting both the synthesis and the intestinal absorption of cholesterol can improve the lipid profile of patients who show a poor response to statin monotherapy better than high dose/high potency statin monotherapy [28-30]. It has been suggested that reducing cholesterol absorption using ezetimibe treatment combined with a statin, which lowers hepatic cholesterol synthesis, may be a practical approach to intensive lipid management and goal achievement compared with treatments that reduce synthesis alone [28-30]. This may be of particular relevance in T2DM patients, who have been shown to have higher levels of NPC1L1 mRNA and increased intestinal absorption of biliary and newly synthesized cholesterol. One mechanism by which NPC1L1 may be increased in T2DM patients is through elevated glucose concentrations [31]. Cultured Caco-2/15 cells exposed to high glucose levels displayed a significant increase in protein expression of NPC1L1, and when ezetimibe was added to the culture medium, the action of the glucose was reduced [31]. In addition to differences in NPC1L1 protein expression, a disturbance in the ATP binding cassette (ABC) proteins G5 and G8, which regulate cholesterol homeostasis, may play a role in the dyslipidemia of diabetic patients. Specifically, compared with non-diabetics, diabetic patients have decreased mRNA expression of both ABCG5 and G8, leading to increased levels of sitosterol and cholesterol in chylomicrons [32]. Taken together, these results suggest that both increased NPC1L1 and lower ABCG5 and G8 may lead to an increase in cholesterol absorption in diabetic patients, and targeting both the synthesis and the intestinal absorption of cholesterol in the treatment of diabetic dyslipidemia may be prudent. This view is further supported by the findings obtained in high cardiovascular risk patients, which demonstrated between-group LDL-C reductions in favor of EZ + simva vs rosuvastatin 10 mg in patients who were not at LDL-C target prior to switch and suggested that there was a high proportion of poor responders to statin therapy in this group [29]. Additional clinical trials to assess cardiovascular outcomes with ezetimibe added to statin therapy are ongoing.

In the present study, both treatment regimens had similar safety and tolerability profiles during the study period. Despite higher baseline laboratory values in the simvastatin group, there were no reports of increased ALT or AST ≥ 3 × ULN nor CK ≥ 5 × ULN in this treatment group during the study, nor were there reports of increases in ALT, AST, or CK in the EZ + simva 10/20 mg group. Accordingly, neither the addition of ezetimibe to simvastatin 20 mg nor doubling the dose of simvastatin to 40 mg resulted in reports of myopathy or rhabdomyolysis. These results are consistent with expectations for these drugs at the doses given and with previous trials in this patient population [17-19,25,33]. Although the incidence of serious adverse events was low, this study was relatively small and not powered nor of sufficient duration to assess the prevalence of rare adverse events. Clinical trial registration: NCT00423488

## Conclusion

In conclusion, the results of this study demonstrate that co-administration of ezetimibe with simvastatin 20 mg may provide a superior alternative for LDL-C lowering compared with doubling the dose of simvastatin to 40 mg in hyperlipidemic patients with T2DM and CHD. In addition, the combination therapy was generally well tolerated, providing an alternative treatment for patients who require further LDL-C reduction than with simvastatin 20 mg alone.

## Competing interests

Funding for the study was provided by Schering-Plough Pharmaceuticals. At the time the study was conducted and this manuscript was written, C.G. was an employee of Schering-Plough Pharmaceuticals. She is now an employee of Merck Sharp & Dohme. G.B., C.B.G., A.E.P. and C.M.R. report no competing interests.

## Authors' contributions

GB, CBG, AEP, and CMR conceived, designed or planned the study and interpreted the results; provided substantive suggestions for revision or critically reviewed subsequent iterations of the manuscript; and provided study materials or patients for the study. CL conceived, designed, or planned the study and interpreted the results; provided substantive suggestions for revision or critically reviewed subsequent iterations of the manuscript. All authors reviewed and approved the final version of the manuscript.

## References

[B1] Expert panel on detection evaluation and treatment of high blood cholesterol in adults Executive Summary of The Third Report of The National Cholesterol Education Program (NCEP) Expert Panel on Detection, Evaluation, And Treatment of High Blood Cholesterol In Adults (Adult Treatment Panel III) JAMA 2001 285 2486 2497 10.1001/jama.285.19.2486 11368702

[B2] Graham I Atar D Borch-Johnsen K Boysen G Burell G Cifkova R Dallongeville J De Backer G Ebrahim S Gjelsvik B Herrmann-Lingen C Hoes A Humphries S Knapton M Perk J Priori SG Pyorala K Reiner Z Ruilope L Sans-Menendez S Op Reimer WS Weissberg P Wood D Yarnell J Zamorano JL Walma E Fitzgerald T Cooney MT Dudina A Vahanian A European guidelines on cardiovascular disease prevention in clinical practice: full text. Fourth Joint Task Force of the European Society of Cardiology and other societies on cardiovascular disease prevention in clinical practice (constituted by representatives of nine societies and by invited experts) Eur J Cardiovasc Prev Rehabil 2007 14 Suppl 2 S1 113 10.1097/01.hjr.0000277983.23934.c9 17726407

[B3] JBS 2: Joint British Societies' guidelines on prevention of cardiovascular disease in clinical practice Heart 2005 91 Suppl 5 v1 52 16365341 10.1136/hrt.2005.079988 PMC1876394

[B4] Assmann G Carmena R Cullen P Fruchart JC Jossa F Lewis B Mancini M Paoletti R Coronary heart disease: reducing the risk: a worldwide view. International Task Force for the Prevention of Coronary Heart Disease Circulation 1999 100 1930 1938 10545439 10.1161/01.cir.100.18.1930

[B5] Haffner SM Lehto S Ronnemaa T Pyorala K Laakso M Mortality from coronary heart disease in subjects with type 2 diabetes and in nondiabetic subjects with and without prior myocardial infarction N Engl J Med 1998 339 229 234 10.1056/NEJM199807233390404 9673301

[B6] Brunzell JD Davidson M Furberg CD Goldberg RB Howard BV Stein JH Witztum JL Lipoprotein management in patients with cardiometabolic risk: consensus statement from the American Diabetes Association and the American College of Cardiology Foundation Diabetes Care 2008 31 811 822 10.2337/dc08-9018 18375431

[B7] Kearney PM Blackwell L Collins R Keech A Simes J Peto R Armitage J Baigent C Efficacy of cholesterol-lowering therapy in 18,686 people with diabetes in 14 randomised trials of statins: a meta-analysis Lancet 2008 371 117 125 10.1016/S0140-6736(08)60761-8 18191683

[B8] Kotseva K Wood D De Backer G De Bacquer D Pyorala K Keil U EUROASPIRE III: a survey on the lifestyle, risk factors and use of cardioprotective drug therapies in coronary patients from 22 European countries Eur J Cardiovasc Prev Rehabil 2009 16 121 137 10.1097/HJR.0b013e3283294b1d 19287307

[B9] Avogaro A Guida P Giorda C Mannucci E Medea G Comaschi M Velussi M Armienti G Zucchetti R The under-use of statin in type 2 diabetic patients attending diabetic clinics in Italy Nutr Metab Cardiovasc Dis 2007 17 32 40 10.1016/j.numecd.2005.12.001 17174224

[B10] Waters DD Brotons C Chiang CW Ferrieres J Foody J Jukema JW Santos RD Verdejo J Messig M McPherson R Seung KB Tarasenko L Lipid treatment assessment project 2: a multinational survey to evaluate the proportion of patients achieving low-density lipoprotein cholesterol goals Circulation 2009 120 28 34 10.1161/CIRCULATIONAHA.108.838466 19546386

[B11] Gotto AM Jr Statins, cardiovascular disease, and drug safety Am J Cardiol 2006 97 3C 5C 16581326 10.1016/j.amjcard.2005.12.005

[B12] Harper CR Jacobson TA The broad spectrum of statin myopathy: from myalgia to rhabdomyolysis Curr Opin Lipidol 2007 18 401 408 10.1097/MOL.0b013e32825a6773 17620856

[B13] van Himbergen TM Matthan NR Resteghini NA Otokozawa S Ai M Stein EA Jones PH Schaefer EJ Comparison of the effects of maximal dose atorvastatin and rosuvastatin therapy on cholesterol synthesis and absorption markers J Lipid Res 2009 50 730 739 10.1194/jlr.P800042-JLR200 19043140 PMC2656667

[B14] Pisciotta L Fasano T Bellocchio A Bocchi L Sallo R Fresa R Colangeli I Cantafora A Calandra S Bertolini S Effect of ezetimibe coadministered with statins in genotype-confirmed heterozygous FH patients Atherosclerosis 2007 194 e116 e122 10.1016/j.atherosclerosis.2006.10.036 17140581

[B15] Davis HR Veltri EP Zetia: inhibition of Niemann-Pick C1 Like 1 (NPC1L1) to reduce intestinal cholesterol absorption and treat hyperlipidemia J Atheroscler Thromb 2007 14 99 108 17587760 10.5551/jat.14.99

[B16] Merck/Schering-Plough Pharmaceuticals Zetia (ezetimibe) [package insert] 2008 North Wales, PA, Merck/Schering-Plough Pharmaceuticals

[B17] Merck/Schering-Plough Pharmaceuticals Vytorin (ezetimibe/simvastatin) [package insert] 2009 North Wales, PA, Merck/Schering-Plough Pharmaceuticals USA

[B18] Gaudiani LM Lewin A Meneghini L Perevozskaya I Plotkin D Mitchel Y Shah S Efficacy and safety of ezetimibe co-administered with simvastatin in thiazolidinedione-treated type 2 diabetic patients Diabetes Obes Metab 2005 7 88 97 10.1111/j.1463-1326.2004.00420.x 15642080

[B19] Goldberg RB Guyton JR Mazzone T Weinstock RS Polis A Edwards P Tomassini JE Tershakovec AM Ezetimibe/simvastatin vs atorvastatin in patients with type 2 diabetes mellitus and hypercholesterolemia: the VYTAL study Mayo Clin Proc 2006 81 1579 1588 10.4065/81.12.1579 17165637

[B20] Constance C Westphal S Chung N Lund M McCrary SC Johnson-Levonas AO Massaad R Allen C Efficacy of ezetimibe/simvastatin 10/20 and 10/40 mg compared with atorvastatin 20 mg in patients with type 2 diabetes mellitus Diabetes Obes Metab 2007 9 575 584 10.1111/j.1463-1326.2007.00725.x 17451425

[B21] Roeters van Lennep HW Liem AH Dunselman PH Dallinga-Thie GM Zwinderman AH Jukema JW The efficacy of statin monotherapy uptitration versus switching to ezetimibe/simvastatin: results of the EASEGO study Curr Med Res Opin 2008 24 685 694 10.1185/030079908X273273 18226326

[B22] Friedewald WT Levy RI Fredrickson DS Estimation of the concentration of low-density lipoprotein cholesterol in plasma, without use of the preparative ultracentrifuge Clin Chem 1972 18 499 502 4337382

[B23] Barrios V Amabile N Paganelli F Chen JW Allen C Johnson-Levonas AO Massaad R Vandormael K Lipid-altering efficacy of switching from atorvastatin 10 mg/day to ezetimibe/simvastatin 10/20 mg/day compared to doubling the dose of atorvastatin in hypercholesterolaemic patients with atherosclerosis or coronary heart disease Int J Clin Pract 2005 59 1377 1386 10.1111/j.1368-5031.2005.00714.x 16351668

[B24] Stein E Stender S Mata P Sager P Ponsonnet D Melani L Lipka L Suresh R Maccubbin D Veltri E Achieving lipoprotein goals in patients at high risk with severe hypercholesterolemia: efficacy and safety of ezetimibe co-administered with atorvastatin Am Heart J 2004 148 447 455 10.1016/j.ahj.2004.03.052 15389231

[B25] Conard S Bays H Leiter LA Bird S Lin J Hanson ME Shah A Tershakovec AM Ezetimibe added to atorvastatin compared with doubling the atorvastatin dose in patients at high risk for coronary heart disease with diabetes mellitus, metabolic syndrome or neither Diabetes Obes Metab 2010 12 210 218 10.1111/j.1463-1326.2009.01152.x 20151997

[B26] Simons L Tonkon M Masana L Maccubbin D Shah A Lee M Gumbiner B Effects of ezetimibe added to on-going statin therapy on the lipid profile of hypercholesterolemic patients with diabetes mellitus or metabolic syndrome Curr Med Res Opin 2004 20 1437 1445 10.1185/030079904X2321 15383192

[B27] Brudi P Vaverkova H Farnier M Averna M Viigimaa M Dong Q Shah A Johnson-Levonas AO Missault L Lipid-Altering Efficacy of Ezetimibe/Simvastatin 10/20 mg Compared with Rosuvastatin 10 mg in High-Risk Patients with and Without Type 2 Diabetes Mellitus (T2DM) [abstract] J Clin Lipidology 2009 3 233 10.1016/j.jacl.2009.04.042

[B28] Conard SE Bays HE Leiter LA Bird SR Rubino J Lowe RS Tomassini JE Tershakovec AM Efficacy and safety of ezetimibe added on to atorvastatin (20 mg) versus uptitration of atorvastatin (to 40 mg) in hypercholesterolemic patients at moderately high risk for coronary heart disease Am J Cardiol 2008 102 1489 1494 10.1016/j.amjcard.2008.09.075 19026302

[B29] Farnier M Averna M Missault L Vaverkova H Viigimaa M Massaad R Vandormael K Johnson-Levonas AO Brudi P Lipid-altering efficacy of ezetimibe/simvastatin 10/20 mg compared with rosuvastatin 10 mg in high-risk hypercholesterolaemic patients inadequately controlled with prior statin monotherapy - The IN-CROSS study Int J Clin Pract 2009 63 547 559 10.1111/j.1742-1241.2009.02022.x 19222610

[B30] Leiter LA Bays H Conard S Bird S Rubino J Hanson ME Tomassini JE Tershakovec AM Efficacy and safety of ezetimibe added on to atorvastatin (40 mg) compared with uptitration of atorvastatin (to 80 mg) in hypercholesterolemic patients at high risk of coronary heart disease Am J Cardiol 2008 102 1495 1501 10.1016/j.amjcard.2008.09.076 19026303

[B31] Ravid Z Bendayan M Delvin E Sane AT Elchebly M Lafond J Lambert M Mailhot G Levy E Modulation of intestinal cholesterol absorption by high glucose levels: impact on cholesterol transporters, regulatory enzymes, and transcription factors Am J Physiol Gastrointest Liver Physiol 2008 295 G873 G885 10.1152/ajpgi.90376.2008 18772361

[B32] Lally SE Owens D Tomkin GH Sitosterol and cholesterol in chylomicrons of type 2 diabetic and non-diabetic subjects: the relationship with ATP binding cassette proteins G5 and G8 and Niemann-Pick C1-like 1 mRNA Diabetologia 2007 50 217 219 10.1007/s00125-006-0504-0 17102949

[B33] Merck & Co I Zocor (simvastatin) tablets [package insert] 2008 Whitehouse Station, NJ, Merck & Co. Inc USA(Package Circular)

